# Perivascular Adipose Tissue as a Target for Antioxidant Therapy for Cardiovascular Complications

**DOI:** 10.3390/antiox9070574

**Published:** 2020-07-02

**Authors:** Andy W. C. Man, Yawen Zhou, Ning Xia, Huige Li

**Affiliations:** Department of Pharmacology, Johannes Gutenberg University Medical Center, 55131 Mainz, Germany; wingcman@uni-mainz.de (A.W.C.M.); yawezhou@uni-mainz.de (Y.Z.); xianing@uni-mainz.de (N.X.)

**Keywords:** perivascular adipose tissue, oxidative stress, sirtuin 1, endothelial nitric oxide synthase, metabolic diseases, cardiovascular diseases

## Abstract

Perivascular adipose tissue (PVAT) is the connective tissue surrounding most of the systemic blood vessels. PVAT is now recognized as an important endocrine tissue that maintains vascular homeostasis. Healthy PVAT has anticontractile, anti-inflammatory, and antioxidative roles. Vascular oxidative stress is an important pathophysiological event in cardiometabolic complications of obesity, type 2 diabetes, and hypertension. Accumulating data from both humans and experimental animal models suggests that PVAT dysfunction is potentially linked to cardiovascular diseases, and associated with augmented vascular inflammation, oxidative stress, and arterial remodeling. Reactive oxygen species produced from PVAT can be originated from mitochondria, nicotinamide adenine dinucleotide phosphate (NADPH) oxidases, and uncoupled endothelial nitric oxide synthase. PVAT can also sense vascular paracrine signals and response by secreting vasoactive adipokines. Therefore, PVAT may constitute a novel therapeutic target for the prevention and treatment of cardiovascular diseases. In this review, we summarize recent findings on PVAT functions, ROS production, and oxidative stress in different pathophysiological settings and discuss the potential antioxidant therapies for cardiovascular diseases by targeting PVAT.

## 1. Introduction

Perivascular adipose tissue (PVAT) is the ectopic fat depot that surrounds large arteries and veins, small and resistance vessels, and skeletal muscle microvessels [1]. In recent decades, PVAT has been revealed as an important endocrine tissue that maintains vascular homeostasis. The role of PVAT in vascular function was first described by the observation that PVAT diminishes agonists-induced contractile responses in the Sprague–Dawley rat aortae in vitro [2]. PVAT exerts an anticontractile effect on vessels in both rodents and humans [3,4]. It is currently known that PVAT regulates vascular function via endocrine and paracrine mechanisms by releasing various factors, including adipokines, cytokines/chemokines, reactive oxygen species, nitric oxide, and hydrogen sulphide (H_2_S) [1]. These factors may enter the media and reach the endothelial layer of blood vessels either by direct diffusion or via the vasa vasorum or the small media conduit networks connecting the medial layer with the underlying adventitia [5,6,7]. These adipose tissue-secreted factors include both proinflammatory and anti-inflammatory vasoactive molecules. These PVAT-derived factors modulate various complex processes, including vascular inflammation and oxidative stress, vascular tone, and smooth muscle proliferation and migration [8,9].

Obesity, with respect to its growing frequency worldwide, has become a major public health concern and a burden to developed countries [10]. Obesity is also known as a critical risk factor for most cardiovascular diseases [11]. Accumulating data from both humans and experimental animal models suggests that the dysfunction of PVAT is involved in obesity-related cardiovascular complications, such as endothelial dysfunction, atherosclerosis, or hypertension [12,13,14]. An ‘obesity triad’ consisting of PVAT hypoxia, inflammation, and oxidative stress is proposed as the central mechanism in obesity-induced PVAT dysfunction [1]. Among the triad, vascular oxidative stress is an important pathophysiological event in cardiometabolic complications, including obesity, type 2 diabetes, and hypertension. During oxidative stress, the production of oxidants (e.g., reactive oxygen species, ROS) exceeds antioxidant defense mechanisms, leading to a redox imbalance [15]. In normal conditions, homeostatic ROS play a critical role as secondary messengers in various intracellular signaling pathways in both innate and adaptive immune responses [16]. ROS from PVAT can originate from the mitochondria, nicotinamide adenine dinucleotide phosphate (NADPH) oxidase, and uncoupled endothelial nitic oxide synthase (eNOS) [15]. An abnormal generation of ROS by PVAT emerges as a potential pathophysiological mechanism underlying vascular injury. The anticontractile effect of PVAT is attenuated after the augmentation of oxidative stress [4]. Therefore, PVAT could be a potential therapeutic antioxidant target for the prevention and treatment of cardiovascular diseases. In this review, we summarize recent findings on PVAT functions, ROS production, and oxidative stress in different pathophysiological settings, and discuss the potential antioxidant treatment of cardiovascular diseases by targeting PVAT.

## 2. PVAT Modulates Vascular Function

The vascular wall of blood vessels is composed of three layers: tunica adventitia, tunica media, and tunica intima. The tunica intima, the inner layer, is a single layer of flattened, polygonal endothelial cells that rest on basal lamina and loose connective tissues, while the tunica media (especially in arteries) mainly consists of vascular smooth muscle cells (VSMC). The adventitia mainly contains connective tissue [17]. PVAT can be found outside the adventitial layer surrounding most of the systemic blood vessels, including large arteries and veins, small and resistance vessels, and skeletal muscle microvessels. Other microvasculature and the cerebral vasculature are free of PVAT [5,8].

The endothelium has long been recognized as an important regulator of vascular tone by releasing vasoactive factors that modulate VSMC contractility. Endothelium can release both vasodilating (such as endothelium-derived hyperpolarizing factors (EDHF), prostaglandin (PGI_2_), and nitric oxide (NO)) and vasoconstricting factors (such as endothelin-1 (ET-1) and thromboxane A2 (TXA2)). The endothelium can control the vascular tone by balancing the release of these vasoactive molecules [18]. It is well known that endothelium dysfunction is a major factor in the pathogenesis of many cardiovascular diseases, including hypertension and atherosclerosis [19], as well as metabolic syndrome and diabetes [20,21]. The importance of the endothelium in vascular function is undebatable; however, it is now known that it is not the sole significant regulator of the vascular tone.

**Figure 1:**
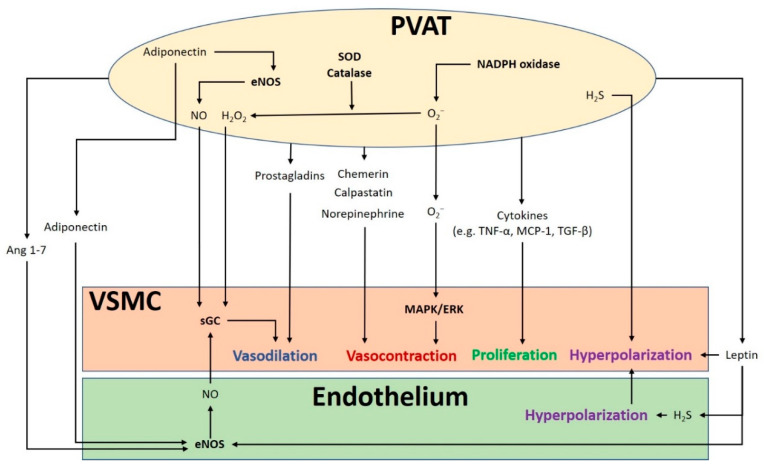
The crosstalk between perivascular adipose tissue (PVAT) and the blood vessel modulates vascular function. PVAT releases vasoactive molecules. H_2_S is synthesized in PVAT and induces vascular smooth muscle cell (VSMC) hyperpolarization. Leptin activates eNOS and stimulates H_2_S production, which lead to endothelium-dependent vasodilatation. H_2_S released from endothelium and PVAT functions as an endothelium-derived hyperpolarizing factor (EDHF) and activates endothelial K^+^ channels. The resulting hyperpolarization of endothelial cells can be transmitted to VSMC. NO and H_2_O_2_ released from PVAT can elicit vasodilatation by activating soluble guanylyl cyclase (sGC). Adiponectin produced by PVAT stimulates NO production in PVAT and in endothelial cells and induces VSMC hyperpolarization. Ang 1–7 acts on endothelial cells and stimulates endothelial NO production. Vasodilating prostaglandins released from PVAT can act on VSMC and stimulate vasorelaxation. Reactive oxygen species (ROS) produced from nicotinamide adenine dinucleotide phosphate (NADPH) oxidases in PVAT can cause contractile response in VSMC via the mitogen-activated protein kinase/extracellular signal-regulated kinase (MAPK/ERK) pathway. Other potential PVAT-derived contracting factors have been suggested, including chemerin, calpastatin, and norepinephrine. Antioxidant enzymes SOD and catalase in PVAT can detoxify ROS to produce H_2_O_2_, which can stimulate vasodilation. Cytokines released from PVAT, including TNF-α, MCP-1 and TGF-β can lead to VSMC proliferation. MAPK/ERK, mitogen-activated protein kinase/extracellular signal-regulated kinase; Ang, angiotensin; eNOS, endothelial nitric oxide synthase; SOD, superoxide dismutase; TNF-α, tumor necrosis factor-α; MCP-1, monocyte chemotactic protein-1; TGF-β, transforming growth factor-β; H_2_S, hydrogen sulphide; VSMC, vascular smooth muscle cells.

The crosstalk between PVAT and the blood vessel is vital for normal vascular function (Figure 1). The modulation of the vascular function by PVAT has been demonstrated by its inhibiting effect on vascular contraction to various agonists in both rodents and humans [3,4]. PVAT exerts anti-contractile function through the release of various PVAT-derived relaxing factors (PVRFs), previously known as the adventitium-derived relaxing factors (ADRFs) [22]. Although it is still unclear how PVRFs exert their anti-contractile effects, a number of potential PVRFs have been suggested, including leptin and adiponectin [23], hydrogen sulphide (H_2_S) [24], hydrogen peroxide (H_2_O_2_) [19], prostaglandins [25,26], NO [27], and angiotensin (Ang) 1–7 [28]. Currently, it is hypothesized that PVAT modulates vascular function through two distinct mechanisms: an endothelium-dependent mechanism through a transferable PVRF and the stimulation of NO release from the endothelium, and an endothelium-independent mechanism through the generation of H_2_O_2_ [27]. The transferable PVRFs modulate the release of endothelial NO and the subsequent activation of potassium (K^+^) channels to facilitate vascular relaxation. On the other hand, the non-transferable anticontractile property of PVAT may involve the generation of H_2_O_2_. A recent study suggests that the mitochondria of PVAT are important sources of O_2_^−^ and H_2_O_2_, which are continuously generated in response to contractile stimuli. PVAT regulates the relaxation and contraction of VSMC [29], while ROS may act as important mediators of PVAT anticontractile effects by direct actions in the VSMC [30].

In addition to PVRF, recent studies have also demonstrated that PVAT can secrete PVAT-derived contracting factors (PVCFs), which modulate vasoconstriction [31,32,33]. Studies in rats have shown that both PVRFs and PVCFs can modulate local vascular tone through endothelium-dependent or endothelium-independent effects, by as-of-yet underdetermined mechanisms [25,34]. Currently, a number of potential PVCFs have been suggested, including chemerin [35], calpastatin [36], norepinephrine (NE) [37], AngII, and ROS [38]. Also, upon perivascular nerve stimulation, PVAT may produce superoxide (O_2_^−^) mediated by NADPH oxidases, which enhances the arterial contractile response. This enhancement of contractile effect involves the activation of tyrosine kinase and the mitogen-activated protein kinase/extracellular signal-regulated kinase (MAPK/ERK) pathway. Moreover, the contractile effects of PVAT are regulated by other antioxidant enzymes including SOD (superoxide dismutase) and catalase. Both Mn-SOD and CuZn-SOD are expressed in PVAT [25,38]. When stimulated by NE, the expression of Mn-SOD is increased and the expression of catalase is decreased in PVAT, which induces the generation of O_2_^−^ in PVAT [30].

It is hypothesized that mitochondria-derived ROS in PVAT modulates vascular reactivity. The uncoupling of mitochondria and the removal of H_2_O_2_ increase the NE-induced contraction in vessel rings surrounded with PVAT [30]. Perivascular nerve activation by electric field stimulation (EFS) increases O_2_^−^ generation in isolated PVAT, which is attenuated by an inhibition of NADPH oxidases. Treatment with apocynin and diphenyleneiodonium chloride (DPI) to inhibit NADPH oxidases attenuates the contractile response to EFS in the vessel rings with PVAT [38]. Also, exogenous O_2_^−^ augments the contractile response to EFS and to phenylephrine in vessel rings without PVAT [38]. Therefore, ROS in PVAT may act as a pivotal signaling molecule in regulating the contraction of VSMC.

A recent study has shown that the anticontractile effects of PVAT can be mediated by vasoactive amines such as dopamine, NE, and serotonin, which can be uptaken and metabolized in PVAT. Monoamine oxidase A/B (MAO-A/B, which catalyzes the oxidative deamination of vasoactive amine) and semicarbazide-sensitive amine oxidase (SSAO, which catalyzes the production of H_2_O_2_ and ammonia) are presented in PVAT, which is responsible for the metabolism of these vasoactive amines. In rat mesenteric arteries, the inhibition of MAO and SSAO, or the inhibition of norepinephrine transporter (NET) with nisoxetine reduced PVAT’s anticontractile effect in response to NE-induced vasocontraction. Furthermore, sympathetic stimulation can trigger the release of adiponectin via β3-adrenoceptor activation in PVAT [39]. Moreover, PVAT can also prevent noradrenaline-induced vasocontraction, by acting as a reservoir of noradrenaline and preventing it from reaching the vessel wall [39]. These studies suggest that PVAT possesses various underdetermined mechanisms, in addition to releasing vasoactive factors, to regulate the vascular function.

## 3. PVAT Contains Both White and Brown Adipose Tissue

PVAT exhibits regional phenotypic and functional differences throughout the vascular system [5,8]. The anti-contractile function of PVAT is determined by the browning and inflammation status. To date, PVAT is known to contain both white (WAT) and brown adipose tissue (BAT). WAT acts mainly as an energy storage [40], while BAT is more vascularized and metabolically active, and mainly associated with thermogenesis [41]. Depending on the vascular bed, PVAT can be WAT-like (e.g., murine mesenteric PVAT), BAT-like (e.g., PVAT of the murine thoracic aorta), or mixed adipose tissue (e.g., PVAT of the murine abdominal aorta) and it possess different vascularization, innervation, and adipokine profiles [5,8,34,42]. The white-to-brown ratio in PVAT is not constant across the vascular bed. For example, in the aorta of rodents, PVAT in the abdominal regions is mainly composed of WAT, while PVAT in the thoracic regions is predominantly BAT [40]. Human coronary PVAT exhibits a histological appearance and gene expression pattern more consistent with WAT than BAT [43]. However, the morphological properties of PVAT in other species are currently less well-defined than murine PVAT.

The thermogenic properties of PVAT have been demonstrated as anti-atherogenic [8]. In mice, PVAT in the thoracic region of aorta has a very low inflammation level even after long-term high-fat diet (HFD) treatment. While PVAT in the thoracic region of aorta is BAT-like, this observation suggests that promoting PVAT browning might have a protective effect on vascular health [41]. Interestingly, PVAT is completely missing throughout the thoracic and abdominal aorta and mesenteric artery of the highly VSMC-selective PPARγ (peroxisome proliferator-activated receptor gamma)-knockout mice [44]. It is suggested that adipocytes in PVAT may potentially share the same smooth muscle protein 22 alpha (SM22α^+^) precursors with VSMC [29]. However, the detailed mechanisms underlying browning or the thermogenesis of PVAT are poorly understood. Nevertheless, mitochondrial biogenesis is important in adipocyte browning [45]. Mitochondrial function is linked to adiponectin production in adipocytes, and enhanced mitochondrial function is needed for adipocyte differentiation as well as ROS generation [46].

## 4. PVAT Dysfunction, Oxidative Stress and Cardiovascular Complications

PVAT dysfunction leads to the imbalance of PVAT-derived vasoactive factors and affects vascular function. There are various physiological conditions and primary mechanisms leading to PVAT dysfunction, including obesity, aging, PVAT remodeling (phenotypic changes of PVAT including an increase in adiposity), an increase in oxidative stress and inflammatory response, and the loss of eNOS and NO [47]. PVAT may shift toward a pro-inflammatory and pro-oxidative state during these complications and may lead to endothelial dysfunction [48]. The generation of ROS including O_2_^−^ and H_2_O_2_ in PVAT is involved in the progression of cardiovascular diseases. PVAT ROS are mostly produced by the mitochondrial oxidative phosphorylation or by the family of NADPH oxidases [38]. Under normal conditions, the deleterious effects of ROS are antagonized by several antioxidant enzyme systems (including catalase, glutathione peroxidase, Mn-SOD, and CuZn-SOD) in PVAT [25,38]. ROS production and lipid peroxidation levels appear to be similar in the PVAT along the aorta, although the relative expression levels of Mn-SOD and CuZn-SOD are different [34]. Considering the importance of ROS on endothelial and PVAT function and the fact that mitochondria are an important source of ROS, modulating mitochondrial function in PVAT is critically important in maintaining the normal anticontractile function. In addition, macrophages in PVAT represent a key modulator of oxidative stress and inflammatory status modulated by oxidative stress. Oxidative stress plays a significant role in promoting interleukin 6 (IL-6) and monocyte chemotactic protein-1 (MCP-1) expression, which lead to the recruitment of monocytes and macrophage in PVAT and the pathology of obesity-induced vascular diseases [49,50,51]. A reduced adiponectin level in PVAT during inflammation and oxidative stress is associated with increased macrophage infiltration [52].

## 5. Obesity-Linked PVAT Dysfunction

Over the past few decades, the prevalence of obesity has doubled worldwide, with a concomitant increase in associated cardiovascular diseases [53]. Obesity has a variety of adverse effects on the cardiovascular system [54]. Although obese patients have a higher risk of developing hypertension, cardiomyopathy, and stroke, endothelial dysfunction is not always evident in in vitro studies. In fact, the dysfunction of PVAT, but not obesity itself, is responsible for obesity-induced vascular disorders. The anticontractile effects of PVAT are attenuated in obese mice compared to lean mice [12,55,56]. When PVAT is removed from the vessel, the anticontractile responses to vasodilators are not different between obese and lean mice, suggesting that obesity does not directly impair the intrinsic vascular bed reactivity but rather the PVAT function [57]. Moreover, mesenteric arteries incubated with PVAT surrounding the thoracic aorta region of aorta from HFD-fed rats show reduced endothelium-dependent relaxation compared to those incubated with aortic PVAT from rats on a standard chow diet [58]. Therefore, it is likely that PVAT dysfunction is related to the development of obesity-associated vascular complications.

**Figure 2:**
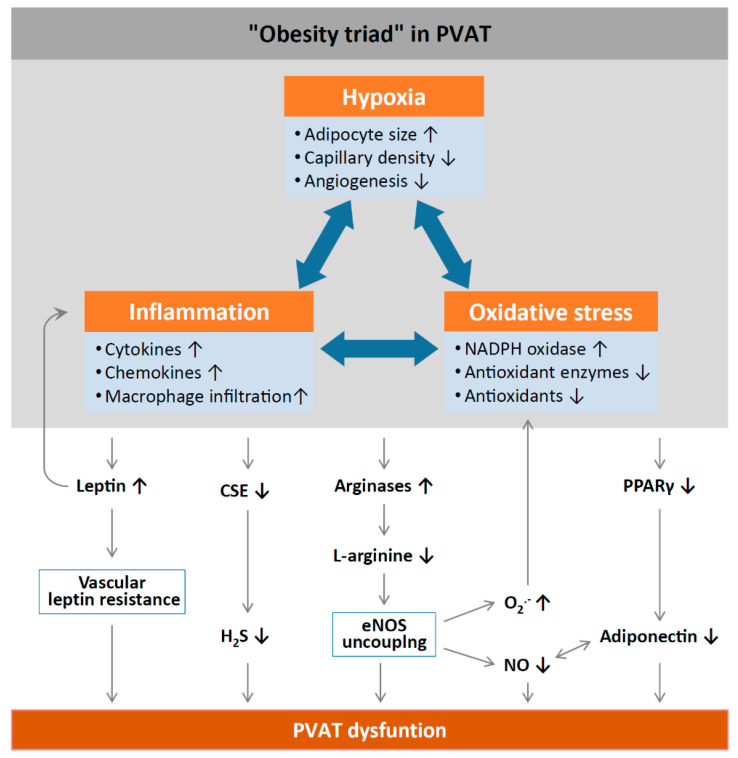
Mechanisms of PVAT dysfunction in diet-induced obesity. High-fat diet (HFD)-induced adipocyte hypertrophy leads to hypoxia and the production of pro-inflammatory cytokines and chemokines, the activation of NADPH oxidases, and the downregulation of antioxidant enzymes (e.g., superoxide dismutase and peroxiredoxin-1) and non-enzymatic antioxidants (e.g., glutathione). Infiltrating immune cells potentiate PVAT inflammation and oxidative stress. Chronic hyperleptinaemia leads to vascular leptin resistance (loss of leptin-induced vasodilatation) and the potentiation of PVAT inflammation. Long-term obesity decreases PVAT H_2_S production by downregulating CSE expression. The upregulation of arginases leads to L-arginine deficiency and eNOS uncoupling (enhanced superoxide production and reduced NO production by eNOS). PVAT adiponectin expression is reduced in obesity, likely due to a downregulation of PPARγ. Normally, NO stimulates adiponectin secretion and adiponectin increases PVAT NO production. This positive feedback mechanism is impaired in obesity. CSE, cystathionine gamma-lyase; H_2_S, hydrogen sulphide; PPARγ, peroxisome proliferator-activated receptors gamma; eNOS, endothelial nitric oxide synthase. Reproduced from Xia et al. 2017 [1] under the terms of the Creative Commons Attribution-Noncommercial License.

Inflammation and oxidative stress in PVAT alter the anticontractile effects under obese conditions (Figure 2). HFD feeding significantly increases the mass of PVAT and the number of hypertrophic adipocytes, and results in WAT characteristics of PVAT in rodents [58]. Obesity may cause inflammation in WAT-like PVAT, characterized by the infiltration of macrophage and dendritic cells with the high expression of inflammatory adipokines and cytokines, including leptin, MCP-1, tumor necrosis factor alpha (TNF-α) [59], and IL-6 [60], while the expression of the anti-inflammatory adipokine adiponectin is reduced in obese PVAT [61]. Obesity-induced PVAT inflammation also stimulates the generation of O_2_^−^ and H_2_O_2_ by NADPH oxidases, which promote aortic wall procontractile activity. The inactivation of O_2_^−^, dismutation of mitochondrial-derived H_2_O_2_, or uncoupling of oxidative phosphorylation can reduce phenylephrine-induced vasocontraction in vessel rings surrounded by PVAT from HFD-fed mice [12,55]. HFD-fed mice also show a reduced expression of SOD3 and glutathione levels in mesenteric PVAT [60]. Also, the anticontractile effects of PVAT are impaired in mice lacking IL-18 specifically in PVAT. Moreover, these mice show increased amount of WAT-like PVAT accompanied with deformed mitochondria and decreased Mn-SOD expression [62]. Obese mice lacking TNF-α receptors in PVAT have reduced H_2_O_2_ generation and sensitivity to phenylephrine-induced vasocontraction, suggesting that oxidative stress significantly contributes to the procontractile shift of PVAT [55]. 

Aldose reductase is an enzyme of the aldoketo reductase super-family that catalyzes the conversion of glucose to sorbitol in the polyol pathway of glucose metabolism, which also depletes the antioxidant glutathione system due to the scavenging of NADPH, thereby increasing the production of ROS [63]. PVAT of diabetic rats exhibits higher levels of markers of oxidative stress including augmented malonaldehyde and aldose reductase activity, which are associated with reduced antioxidant protection [51]. In addition, increased Ang II levels may induce ROS production during obesity in systemic and related adipose tissue including PVAT [64]. eNOS uncoupling and increased O_2_^−^ generation are proposed to contribute to the PVAT-induced endothelial dysfunction in obese mice [56]. The impaired anticontractile effects of PVAT upon HFD feeding are not only dependent on the endothelium but are also a consequence of reduced NO bioavailability due to L-arginine deficiency and eNOS uncoupling in WAT-like PVAT [56,65]. L-arginine supplementation and arginase inhibition could reverse obesity-induced vascular dysfunction ex vivo [56]. In a human study, CuZn-SOD, peroxiredoxin-1, and adiponectin expressions are reduced in obese subjects compared to healthy subjects [65]. The anti-contractility of aortic rings with normal PVAT is improved when incubated with SOD, catalase, or TNF-α, while the anticontractile effect is attenuated when aortic rings with obese PVAT are incubated with anti-TNF-α antibodies or free radical scavengers [65]. 

PVAT inflammation could be related to hypoxia. Hypoxia stimulates the production of inflammatory cytokines and chemokines from PVAT and infiltrating macrophages [4]. In obesity, adipocytes become hypertrophic, leading to inadequate perfusion and consequent local hypoxia. Hypoxia-inducible factor alpha (HIF-1α), a key mediator of hypoxia, is increased in the adipose tissue of obese subjects [66]. HIF-1 α is responsible for the stimulation of inflammatory mediator production, such as TNF-α and IL-6, and for suppressing the expression of adiponectin [67]. Incubation with TNF-α and IL-6 in PVAT leads to the attenuation of the anticontractile effects of PVAT, while the induction of hypoxia also causes inflammation and the loss of the anti-contractile function of PVAT [4]. This hypoxia-induced PVAT dysfunction can be normalized by in vitro incubation with either anti-TNF-α antibody, anti-IL-6 antibody, or by catalase and SOD antagonists. From these observations, PVAT loses its anticontractile properties after the increase of oxidative stress during obesity. In addition, obesity related PVAT dysfunction, increased inflammation, and oxidative stress are critical features in atherogenesis. Atherosclerotic lesions are characterized by the activation of NADPH oxidases, as well as increased eNOS uncoupling and O_2_^−^ generation [68]. The combination of oxidative stress and inflammation creates a vicious cycle that is further supported by a variety of metabolic and genetic risk factors leading to atherogenesis [69]. Therefore, increased oxidative stress in PVAT is a critical link between obesity and vascular complications.

## 6. PVAT Dysfunction and High Sugar Diet

PVAT dysfunction, oxidative stress, and carbohydrates may be particularly related. A fructose-rich diet has been shown to promote endothelial dysfunction and metabolic diseases [70]. One study has demonstrated that fructose can induce the changes in the PVAT lipid profile and the oxidative stress biomarkers which are associated with vascular homeostasis. Fructose feeding leads to the reduced activity of antioxidant enzymes (SOD and glutathione peroxidase) and augments oxidative stress in rat PVAT, which in turn promotes endothelial dysfunction [71]. Aldose reductase, which is involved the polyol pathway of glucose metabolism, is highly expressed in the PVAT from diabetic rats, and is associated with a higher oxidative marker (see above). A recent study shows that a high sugar diet significantly increases ROS generation and promotes pro-oxidative phenotypes in PVAT [72]. In particular, O-GlcNAcylation of eNOS in PVAT is increased by a high sugar diet in rats as well as in hyperglycemic human patients, suggesting that O-GlcNAcylation of eNOS may be involved in the high sugar diet-induced oxidative stress in PVAT [72].

In patients with type 2 diabetes, serum adiponectin levels are lower, and the adiponectin from PVAT are correlated with the increased NADPH-oxidase activity in the arteries [73]. In addition, a recent study suggests that type 2 diabetes is associated with PVAT dysfunction that contributes to oxidative stress and endothelial dysfunction. MnSOD activity is significantly lower in diabetic PVAT, which reduces its ability to eliminate O_2_^−^ [51]. Diabetic PVAT aggravates endothelial dysfunction and exhibits higher levels of markers of oxidative damage such as aldose reductase, and lower levels of antioxidant enzymes. This further supports the idea that obesity-related vascular complications are critically related to oxidative stress-induced PVAT dysfunction.

## 7. Aging and PVAT Dysfunction

The impact of obesity on PVAT is well known, while only a few studies have focused on the effects of aging on PVAT. Aging is another important risk factor for the development of cardiovascular diseases associated with the decline of endothelial function. Endothelial senescence is a key mechanism of aging-related vascular dysfunction [74]. Cellular stressors such as DNA damage, oxidative stress, and metabolic stimuli can activate p53/p21 and p16 tumor suppressor pathways and induce cell cycle arrest [75]. The expressions of p21 and p16 (senescence markers) are significantly increased in the arteries of old mice, and are associated with the oxidative stress-mediated attenuation of NO-dependent endothelial function [76]. However, endothelial senescence itself cannot completely explain the phenotype and progression of vascular aging. In fact, both aging and obesity might affect PVAT in a comparable manner [40].

During aging, structural and functional changes have been observed in vascular tissues. The effect of aging on vascular endothelium and VSMC has been widely investigated, while less is known about the aging of PVAT. In rats, aging attenuates the anti-contractile effects of PVAT surrounding thoracic aorta, and reduces the amount of BAT-like PVAT [77]. Aging exacerbates obesity-induced oxidative stress and inflammation in PVAT, stimulates the secretion of inflammatory factors from PVAT, and affects the phenotypic alterations of the vascular wall in obese mice [59]. Moreover, aging promotes ROS production in PVAT, which subsequently contributes to aging-related vascular injury [77,78]. Elevated levels of a protein inhibitor of activated STAT1 (PIAS1) and lower expression of a signal transducer and activator of transcription 1 (STAT1)- or a nuclear factor ‘kappa-light-chain-enhancer’ of activated B-cells (NFκB)-regulated genes involved in adipocyte differentiation, inflammation, and apoptosis are observed in PVAT surrounding the thoracic aorta of aged mice [79]. Senescence-accelerated mouse prone 8 (SAMP8), a mouse line that has accelerated aging, shows vascular dysfunction with associated hypertension and cognitive decline [80]. In aorta of SAMP8 mice, an increased level of ET-1, inducible nitric oxide synthase (iNOS) and cyclooxygenase 2 (COX-2), oxidative stress markers, and a reduced level of eNOS and COX-1 are detected [81]. Moreover, PVAT has reduced anti-contractile effects in SAMP8 mice, which is also associated with increased oxidative stress and inflammation [81]. The production or activity of adiponectin may contribute to the loss of anti-contractile effects of the aged PVAT surrounding the mesenteric arteries of SAMP8 mice [81]. The differentiation capacities of PVAT-derived stromal cells (PVASCs) are altered during aging. Also, the deletion of PGC-1α (proliferator-activated receptor-gamma and coactivator 1 alpha) in aged PVASCs exacerbates arterial remodeling and attenuates the browning of adipose tissue [82]. A recent study has revealed that the mineralocorticoid receptor (MR) is activated in the mitochondria of PVAT in obese mice, which causes premature-aging in adipose tissue and senescence, resulting in the loss of the anticontractile effects [83]. These results suggest that downregulation of PGC-1α and mitochondrial dysfunction in PVAT may contribute to the aging phenotype.

## 8. PVAT and Arterial Remodeling

Arterial remodeling is known as the active process of structural alteration that is controlled by the crosstalk between the endothelium and VSMC [17]. Arterial remodeling and stiffening can be associated with obesity [84], while in hypertensive patients, low-grade inflammation and hypoadiponectinaemia have a positive association to the detrimental effects on aortic stiffness [85]. Therefore, the contribution of PVAT to arterial remodeling has also been recently explored. It is currently hypothesized that PVAT contributes to vascular remodeling by a multitude of factors including PVAT-derived molecules, extracellular vesicles, and progenitor cells under disease conditions [29]. Indeed, PVAT secretes a spectrum of growth factors, including TGF-β, basic fibroblast growth factor (bFGF), placental growth factor (PLGF), and hepatocyte growth factor (HGF), which are well-known in stimulating VSMC proliferation [86]. Also, inflammatory and oxidative responses in PVAT significantly stimulate VSMC proliferation partly by promoting the expression of matrix metallopeptidases 2 (MMP-2) in PVAT, which promotes that expression of TGF-β in VSMCs [87].

Neointima formation is promoted by the transplantation of adipose tissue from HFD-fed wild-type mice to the carotid artery of immune-deficient mice [88], suggesting that adipose tissues contribute to obesity-induced arterial remodeling. Moreover, conditioned medium from the PVAT of obese rats promotes the proliferation and differentiation of VSMC in vitro [89]. In mice, arterial stiffening is associated with oxidative injury and inflammation, accompanied by immune cell infiltration and T cell activation in the PVAT [90]. Oxidative stress in adipocytes subsequently stimulates the recruitment of immune cells [91], while the contributions of the PVAT to arterial remodeling in pathological conditions could be partly mediated by increased macrophages and T cell infiltration in proinflammatory PVAT.

Interestingly, the removal of PVAT enhances neointima formation upon intravascular injury, while the local delivery of recombinant adiponectin or transplantation of subcutaneous adipose tissue can reduce the neointima formation in the injury area [92]. Moreover, the contribution of adiponectin in preventing arterial remodeling is evidenced by the increased VSMC proliferation and neointimal formation in response to vascular injury in adiponectin-deficient mice [92]. On the other hand, incubation with recombinant leptin or conditioned medium from visceral adipose tissue stimulate VSMC proliferation in vitro [88]. The overexpression of leptin in PVAT of wild-type mice, but not in leptin receptor-deficient mice, can enhance VSMC proliferation and neointima formation [88]. The leptin receptor antagonist also inhibits obese PVAT-induced VSMC phenotypic switching [89]. In obese mice, PVAT induces VSMC phenotypic switching partly by the MAPK signaling pathway, while leptin receptor antagonist can upregulate the phosphorylation of MAPK and attenuate VSMC phenotypic switch [89]. Visfatin, another adipokine mainly expressed in PVAT, can act as a nicotinamide phosphoribosyltransferase and produce nicotinamide mononucleotide, which mediates the VSMC proliferation-activating MAPK/ERK pathway [93]. Adipokines from PVAT may be critical in modulating oxidative stress and arterial remodeling. C1q/TNF-related protein 9 (CTRP9) may contribute to VSMC phenotypic switching by mediating ERK signaling. CTRP9 can suppress the TGF-β1/ERK1/2 pathway and promote apoptosis in response to hypoxia, thus preventing VSMC proliferation [87]. On the other hand, resistin, which is associated with an increased protein kinase C epsilon (PKCε)-dependent expression of MMP, is responsible for promoting the proliferation of VSMC [87]. Arterial remodeling is associated with resistin and related to PKCε- dependent NADPH oxidases activation and ROS generation [94].

During aging, the characteristics of the progenitor cells in PVAT change. Periaortic stem cells from young mice can differentiate into BAT-like adipocytes and inhibit neointima formation, while aged stem cells lose this ability and promote neointima hyperplasia in the injured artery [82]. PVAT may differentiate from neural crest cells which highly express wingless-type MMTV integration site family, member 1 (Wnt1). The deletion of PPARγ mediated by Wnt1-Cre causes the delay and dysplasia of PVAT development in mice [95]. The deletion of PPARγ aggravates Ang II-induced hypertension in these mice compared to wild-type mice. Interestingly, Ang II infusion markedly aggravates arterial remodeling in common carotid arteries but not in the aortic arch of these mice [95]. A recent study also suggests that PVAT secretes extracellular vesicles containing microRNAs and acts as an intercellular message signaler. In obese mice, abundant extracellular vesicles are secreted from PVAT, which evoke inflammatory responses in aorta. MicroRNAs in the extracellular vesicles, such as miR-221-3p, can promote VSMC proliferation and migration [96].

## 9. PVAT as a Potential Target for Antioxidant Treatment

**Figure 3:**
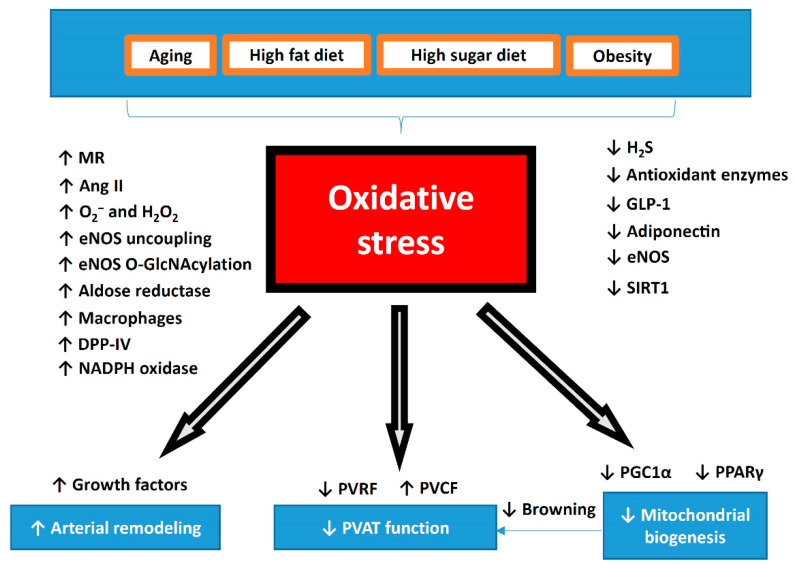
Summary of oxidative stress in the pathophysiology of PVAT dysfunction. During aging or diet-induced obesity, oxidative stress is significantly increased in PVAT. Dysfunctional PVAT promotes cardiovascular diseases via the crosstalk with the underlying vascular cells, including the endothelium and VSMC under physiological and pathological conditions. Reversing the shift of PVAT towards WAT-like during the development of obesity and aging might be a crucial strategy to maintain a healthy vasculature. The potential use of antioxidant or targeting oxidative stress can be novel therapeutic target for PVAT-related cardiovascular complications. MR, mineralocorticoid receptor; Ang II, angiotensin II; DPP-IV, dipeptidyl peptidase-IV; H_2_S, hydrogen sulphide; GLP-1, glucagon-like peptide-1; eNOS, endothelial nitric oxide synthase; SIRT1, sirtuin 1; PVRF, PVAT-derived relaxing factors; PVCF, PVAT-derived contracting factors; PGC-1α, peroxisome proliferator-activated receptor gamma coactivator 1-alpha; PPARγ, peroxisome proliferator-activated receptors gamma.

PVAT-induced vascular dysfunction in the thoracic aorta of obese mice is associated with systemic inflammation and oxidative stress. In addition, PVAT is involved in the modulation of other vascular complications as mentioned above, which are associated with oxidative stress and inflammation in PVAT. The signaling molecules and pathways in PVAT, such as those involving adipokines, H_2_S, glucagon-like peptide 1, or pro-inflammatory cytokines, are among the potential novel pharmacological therapeutic targets of PVAT (Figure 3). Here, we summarize recent studies on antioxidant targets and treatments on PVAT.

In HFD-fed rats, the administration of ethanolic extract of Mangosteen pericarp (EEMP) that contains xanthone as an antioxidant can normalize the thickened PVAT and reduce the expression of VCAM-1 to prevent arterial remodeling [97]. Also, long-term treatment with melatonin in mice models of accelerated aging can increase the expression of vasculoprotective markers, decrease oxidative stress and inflammation, and normalize the anti-contractile effects of PVAT [81]. Polysaccharide peptides contain D-Glucan, a bioactive substance which possesses anti-inflammatory and antioxidant properties. Recent studies have shown that polysaccharide peptides isolated from fungi can normalize the H_2_O_2_ level in PVAT by inducing the expression of SOD and catalase in HFD fed-rat, thus preventing PVAT thickening and arterial remodeling [98,99].

However, a recent study shows that a diet devoid of genistein, an isoflavone, can reduce superoxide production and iNOS expression in PVAT surrounding the mesenteric artery in obese mice. In obese mice, genistein supplementation prevents weight gain but promotes vascular oxidative stress, which is attributed to a decline in SOD activity and PVAT iNOS expression [100,101]. Similarly, vitamin E supplementation can decrease oxidative stress and reduce collagen deposition, but not macrophage infiltration in adipose tissue [102]. These results suggest that antioxidant supplementations warrant additional studies to investigate the detailed mechanisms and targets to modulate PVAT function.

### 9.1. Improving eNOS Functionality

PVAT dysfunction is causally linked to vascular dysfunction and can be normalized by restoring and enhancing PVAT eNOS function. Indeed, in PVAT, both adipocytes and endothelial cells of the capillaries and vasa vasorum are stained positive for eNOS in immunohistochemistry analyses. In addition, NO production can be directly visualized in PVAT adipocytes in situ with fluorescence imaging techniques [103], and PVAT contributes to vascular NO production [104]. Moreover, recent studies have reported the gene and protein expression of eNOS in PVAT [56,105]. However, eNOS expression may vary in the PVAT along different regions of the vasculature. Abdominal PVAT has a lower eNOS expression compared with thoracic PVAT, while eNOS expression in the vessel walls are similar in both the abdominal and thoracic regions [34]. In addition, L-NAME enhances the phenylephrine-induced contraction in endothelial-denuded rings with PVAT from thoracic but not abdominal aorta [34]. These results suggest that the function of PVAT may vary along different regions of the vessel due to the differential expression of eNOS.

Reduced eNOS expression is observed in the PVAT of diet-induced obese rats [106] and mice [60]. Currently, there are at least three well-known mechanisms that contribute to the reduced eNOS activity and function in PVAT, including a lack of L-arginine, a reduction in eNOS serine 1177 phosphorylation, and enhanced eNOS acetylation. In obese mice, the upregulation of arginase [56] and the increased acetylation of eNOS [107] reduce NO production and lead to eNOS uncoupling [108]. An uncoupled eNOS produces superoxide at the expense of NO [109,110] and thus contributes to oxidative stress in PVAT [56]. In *low-density lipoprotein receptor* (*Ldlr*) knockout mice, the thoracic aortic PVAT shows enhanced eNOS expression and NO levels, while it also protects against impaired vasorelaxation to acetylcholine and insulin. This suggests the protective role of PVAT in enhancing eNOS expression and improving endothelial function [105].

Standardized *Crataegus* extract WS^®^ 1442, with antioxidative properties, can restore the vascular function in the PVAT-containing aorta of HFD-fed mice without any effects on body weight or fat mass [107]. WS^®^ 1442 treatment reverses the reduced phosphorylation of Akt (protein kinase B) and eNOS, as well as the enhanced acetylation of eNOS in PVAT. On the other hand, obesity-linked PVAT and endothelial dysfunction are also associated with altered prostaglandin production and impaired K^+^ channel activation [106]. As mentioned, HFD-induced PVAT dysfunction is associated with increased leptin levels and a reduction of eNOS and NO production [57]. Obesity-linked PVAT dysfunction is also associated with AMP-activated protein kinase (AMPK) phosphorylation [111]. Moreover, plasma adiponectin levels and adiponectin expression in the adipose tissue are decreased in *eNOS* knockout mice [112]. Long-term adiponectin treatment in HFD-fed rats can normalize NO-dependent vasorelaxation partly by enhancing the phosphorylation of eNOS in the endothelium of mesenteric arteries [113]. Recently, a study has shown that treatment with methotrexate, an anti-inflammatory drug with antioxidant effects, can improve PVAT/endothelial dysfunction and ameliorate adipokine dysregulation via the activation of the AMPK/eNOS pathway [114]. In addition, eNOS-derived NO can promote adiponectin synthesis and mitochondrial biogenesis [115]. eNOS is abundantly expressed in both BAT and isolated brown adipocytes [116], suggesting that PVAT eNOS could also facilitate browning or the thermogenesis of PVAT. Therefore, eNOS-mediated PVAT adaptive thermogenesis may be targeted for improving PVAT function.

A recent study suggests that aerobic exercise training upregulates the expression of anti-oxidant enzymes in PVAT and decreases oxidative stress with beneficial effects on endothelium-dependent vasorelaxation [117]. Aerobic exercise training stimulates angiogenesis in adipose tissue and PVAT, which improves blood flow, reduces hypoxia and macrophage infiltration [118], and improves vascular function [119]. The beneficial effects of exercise training may be attributed to the normalization of eNOS activity [120] or the reduction of iNOS expression in PVAT [121]. Exercise training can increase eNOS and phospho-eNOS expression in both the vascular wall and the PVAT, as well as increase adiponectin in the PVAT and reduce ROS in the vascular wall [120]. Sustained weight loss in rats restores eNOS expression and improves PVAT NO production [106].

### 9.2. Restoring Brown-Like PVAT

Reversing the white features of PVAT to brown characteristics or maintaining PVAT beige features might be a crucial strategy to maintaining a healthy vasculature. As previously mentioned, PVAT displays phenotypic heterogeneity according to its locations along the vascular system. PVAT surrounding larger blood vessels is BAT-like, while it is WAT-like in areas surrounding smaller blood vessels. The gradual changes into WAT-like characteristics of PVAT during obesity and aging are associated with the alteration of the PVAT secretome profile, including those factors involved in the regulation of vascular tone, blood pressure, and arterial remodeling [59]. BAT-like PVAT could prevent inflammation and oxidative stress under physiological conditions, while WAT-like PVAT is accompanied by augmented inflammation and oxidative stress and reduced NO bioavailability under obese conditions. The induction white-to-brown transition of white-like PVAT might be associated with reduced oxidative stress. Brown PVAT induces cyclic guanosine monophosphate (cGMP)-dependent protein kinase G type-1α activation, via NADPH oxidase 4 (Nox4)-derived H_2_O_2,_ and reduces vascular contractility [122].

There are currently a few strategies that are able to induce browning in WAT, including cold challenge or the application of growth factors such as FGF21 [123], atrial natriuretic peptide (ANP) [124], and bone morphogenetic proteins (BMP) [125]. It is hypothesized that browning of adipose tissue is beneficial in preventing obesity and its associated cardiovascular diseases [126]. Targeting the restoration of BAT-like characteristics in PVAT might be a strategy to maintain the homeostasis of blood vessels and prevent PVAT dysfunction-related vascular complications.

Cold acclimation is a well-known stimulus to induce the browning process of adipose tissue. Upon cold acclimation, PVAT attenuates age-dependent and HFD-induced endothelial dysfunction and atherosclerosis in mice, which is associated with decreased pro-inflammatory markers [127]. Cold exposure can also stimulate the browning effect on abdominal aortic PVAT in HFD-fed rats by increasing uncoupling protein 1 (UCP-1) and PGC-1α expression levels. Expression levels of TNF-α, IL-6, and p65 are significantly reduced, while phospho-AMPK expression is increased in PVAT with cold exposure [128]. In addition, cold conditions stimulate glucose uptake and triglyceride clearance in adipose tissues, which may also contribute to the modulation of oxidative stress in PVAT [129,130]. MitoNEET is a mitochondrial membrane protein that is regulated by thermogenic genes such as PGC-1α and can be upregulated by cold exposure in PVAT. The overexpression of mitoNEET in PVAT significantly prevents arterial stiffness and atherosclerosis [131,132]. Therefore, potential mitoNEET ligands, including rosiglitazone and resveratrol, can be used to target mitochondrial biogenesis [133].

In addition to cold acclimation, exercise training also induces a shift to a BAT-like characteristic and thermogenic response, which is associated with enhanced eNOS expression and reduced oxidative stress in rat PVAT [134]. This suggests that aerobic exercise training and weight loss are beneficial to cardiovascular health via modulating eNOS expression and browning in PVAT.

### 9.3. Sirtuin 1 (SIRT1)

SIRT1 plays a pivotal role in modulating the browning process, protecting against vascular injury by reducing local superoxide production, and enhancing adipokines production in PVAT [135]. In mice knocking-down of SIRT1 specifically in adipose tissue, the obesity-induced brown-to-white transition of PVAT is exaggerated in vivo and endothelial dysfunction is accelerated [135].

In obese mice, treatment with the SIRT1 specific activator SRT1720 could prolong the lifespan and reverse HFD-induced organ damage via normalizing the acetylation of PGC-1α in adipose tissue [136]. Moreover, activation of SIRT1 abolishes dysregulated PVAT adipokine release after inflammatory insult [137] and reduces inflammatory cytokines release which promotes arterial remodeling in aged mice [138]. On the other hand, resveratrol can ameliorate adipokine release from dysregulated PVAT by the SIRT1/AMPK pathway [139]. SIRT1 also regulates the secretion of adiponectin in PVAT through the interaction with forkhead box protein O1 (FOXO1) [139]. The SIRT1 activator resveratrol can also normalize oxidative stress-induced cytokine release from PVAT and prevent arterial remodeling in aged mice [140].

Recent studies have provided evidence that resveratrol improves PVAT function [137,139,141]. The activation of the SIRT1/AMPK signaling in PVAT can regulate adipokine expression, ameliorate endothelial dysfunction caused by inhibiting NFκB activation, and alter PVAT inflammation induced by fructose- [141] or HFD-feeding [137]. The oxidative stress in PVAT may lead to increased pro-inflammatory cytokine and chemokine secretion, and the superoxide derived from PVAT promotes artery stiffening in aged mice [138]. Resveratrol can also alleviate oxidative stress-induced cytokine release from PVAT, and subsequently improve arterial wall hypertrophy and adventitial collagen I accumulation to prevent arterial stiffening in aged mice [140]. Resveratrol or other anti-oxidants may improve PVAT function by scavenging the superoxide and normalizing the expression of TNF-α, IL-6, MCP-1, adiponectin, PPARγ, and eNOS phosphorylation in PVAT [141].

In addition, the activation of SIRT1 promotes mitochondrial biogenesis in adipose tissue via PGC-1α [142] and is involved in adipose tissue browning [143], suggesting that SIRT1 may also be involved in the regulation of PVAT browning. On the other hand, NO can regulate SIRT1 expression in adipose tissues [144], and it is conceivable that NO may also regulate SIRT1 in PVAT. Therefore, the interplay between PVAT SIRT1 and eNOS in controlling the browning and inflammation status of PVAT has been recently proposed [145]. SIRT1 may be a critical target in maintaining browning and normal adipokines secretion in PVAT.

### 9.4. PPARγ and PGC-1α

PPARγ and PGC-1α are important targets to rescue mitochondrial and PVAT function by modulating the white-to-brown differentiation of adipocytes. The highest levels of *PPARγ* are expressed in adipose tissue [146]. Important antioxidant enzymes including heme oxygenase-1 (HO-1) and nuclear factor erythroid 2-related factor 2 (Nrf2)/are regulated through the PPARγ pathway [147]. The inactivation of PPARγ exaggerates ET-1-induced vascular injury, suggesting a protective role of PPARγ in cardiovascular diseases through the modulation of pro-oxidant and proinflammatory pathways [148]. In a rat model of obesity and metabolic syndrome, treatment with the PPARγ agonist (rosiglitazone) increases insulin sensitivity, reduces fasting insulin levels and triglyceride concentration, increases CSE expression and activity as well as PVAT H_2_S production, and is associated with improvements in the anticontractile effect of PVAT on aortic rings [149]. Liraglutide treatment enhances PGC-1α and UCP1 expression in PVAT from HFD mice. [150]

### 9.5. Adipokines

Adipokines are linked to insulin resistance, oxidative stress, inflammation, and immune response [13]. Treatment with irisin, an adipokine, improves glucose and lipid metabolism, reduces plasma levels of TNF-α and malondialdehyde, and increases plasma adiponectin levels in obese mice. Moreover, irisin treatment can normalize the reduced anti-contractile properties of aortic PVAT in obese mice [151]. The beneficial effects of irisin are associated with the upregulation of the HO-1/adiponectin in PVAT and the browning of PVAT, as well as reduced superoxide production and TNF-α expression [151].

PVAT-derived adiponectin can normalize endothelial function, partly by enhancing eNOS phosphorylation in the endothelium [113]. The exposure of adipocytes to ROS such as H_2_O_2_ results in a reduction in adiponectin expression [152]. Adiponectin plays a central role in mediating crosstalk between vascular and adipose tissue. Through paracrine mechanisms, adiponectin from PVAT adipocytes regulates NO production in adjacent adipocytes [153]. Adiponectin released from PVAT induces vasorelaxation and reduces vascular oxidative stress by inhibiting NADPH oxidases activity. On the other hand, vascular oxidative stress can upregulate adiponectin expression via PPARγ activation in PVAT [73]. Capsicin, a spicy anti-oxidant component of hot peppers, can modulate the expression of adipokines and ameliorate obesity-induced adipose tissue dysfunction, as well as prevent macrophage infiltration into adipose tissue in obese mice [154]. These suggest that antioxidant compounds can be used to modulate the expression of adipokines and target PVAT dysfunction.

### 9.6. GLP-1 and DPP-IV

Glucagon-like petide-1 (GLP-1) has been demonstrated to have cardiovascular protective effects [155] and to improve endothelial function in obesity [156]. Recently, GLP-1 has been demonstrated to activate genes related to fatty acid oxidation and insulin signaling pathways, thus enhancing antioxidant capacity. The Nrf2/HO-1 antioxidant pathway is induced by oxidative stress in PVAT [151]. Liraglutide, a GLP-1 receptor agonist with an antioxidant property, has been shown to alleviate vascular dysfunction by modulating the protein kinase A (PKA)-AMPK-PGC-1α pathway and enhancing antioxidant enzymatic system Nrf2/HO-1 in PVAT in HFD-induced obese mice. It is suggested that liraglutide enhances the HO-1/adiponectin axis and alleviates HFD-induced oxidative stress in PVAT [150].

In addition, dipeptidyl peptidase IV (DPP-IV) is an adipokine with potential relevance in cardiovascular disease, and various DPP-IV inhibitors have been shown to exert direct antioxidant effects in rat and mice [157,158]. Indeed, DPP-IV degrades GLP-1 [159], and has been suggested as a pathophysiological link between obesity and the metabolic diseases. The administration of teneligliptin, a DPP-IV inhibitor, results in a variety of beneficial effects including alleviating inflammation and oxidative stress in both the vasculature and PVAT, thus reducing atherosclerosis progression in *apolipoprotein E* (*ApoE*) knockout mice [160]. Recently, two polyphenolic compounds (coumarin and quercetin) have been suggested to inhibit DPP-IV [161]; however, further studies are needed to investigate whether polyphenolic compounds may reduce oxidative stress and target DPP-IV in PVAT.

### 9.7. Renin-Angiotensin System (RAS) Inhibitors

Oxidative stress is closely correlated to the renin-angiotensin system (RAS). Ang II is a potent inducer of ROS, and adipose tissue Ang II levels are increased during obesity. Ang II mediates the PVAT-associated increase of contractile response to perivascular neuronal excitation [162]. Adipose tissue RAS is involved in the control of adipogenesis and adipose tissue mass [163]. The secretion of angiotensinogen is reduced in ROS-treated adipose tissue in vitro and in obese mice, while treatment with an antioxidant N-acetyl cysteine can normalize the angiotensinogen secretion [164]. In rat mesenteric arteries, treatment with either an ACE inhibitor (enalaprilat) or Ang II type 1 receptor antagonist (candesartan) reduces the PVAT-mediated potentiation of PVAT superoxide-induced vasocontraction [162]. In addition, chronic treatment with an ACE inhibitor (quinapril) reduces blood pressure and alleviates the potentiation effects of PVAT superoxide-induced contractions [162]. Sulphhydrylated ACE inhibitor (S-zofenopril) improves vascular function by the dual-action of inhibiting ACE and potentiating the H_2_S pathway in spontaneous hypertensive rats [165].

### 9.8. H_2_S

Exogenous H_2_S administration can inhibit ROS production and suppress vascular oxidative stress in hypertensive rats [166]. The protective effect of H_2_S in suppressing vascular oxidative stress may be attributed to the inhibition of angiotensin II receptor type 1 action, the downregulation of NADPH oxidases, as well as the upregulation of antioxidant enzymes [167]. H_2_S can also be modulated by currently used cardiovascular medications. Atorvastatin has been shown to improve PVAT function in spontaneously hypertensive rats [168]. Treatment with lipophilic atorvastatin increases the PVAT H_2_S levels and inhibits mitochondrial oxidation, which promotes the anticontractile effect of PVAT [24]. Atorvastatin leads to the decrease in coenzyme Q level, which is a cofactor of H_2_S oxidation by sulphide:quinone oxidoreductase, resulting in the inhibition of the H_2_S metabolism and an increase in the H_2_S level. Other than H_2_S, statins do not impair mitochondrial oxidation of organic substrates [169]. In addition, cannabinoid receptor type 1 (CB_1_) agonists inhibit H_2_S mitochondrial oxidation leading to elevated H_2_S levels in PVAT [149]. The imidazoline I_1_ receptor agonist moxonidine has also been shown to increase H_2_S production in rats [170].

## 10. Conclusions and Future Directions

**Figure 4:**
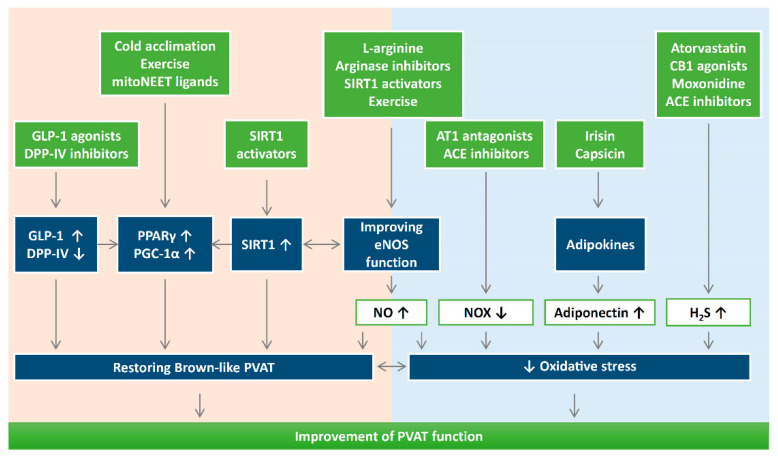
The potential use of antioxidants or targeting oxidative stress can be novel therapeutic targets for PVAT-related cardiovascular complications. It is important to maintain a physiological ROS level for adipogenic differentiation. Reducing oxidative stress and reversing the shift of PVAT towards WAT-like during the development of obesity and aging might be a crucial strategy to improve PVAT function. Detailed research is needed to dissect the molecular mechanisms of how oxidative stress modulates mitochondrial biogenesis in PVAT. DPP-IV, dipeptidyl peptidase-IV; H_2_S, hydrogen sulphide; GLP-1, glucagon-like peptide-1; eNOS, endothelial nitric oxide synthase; SIRT1, sirtuin 1; PGC-1α, peroxisome proliferator-activated receptor gamma coactivator 1-alpha; PPARγ, peroxisome proliferator-activated receptors gamma; NOX, nicotinamide adenine dinucleotide phosphate oxidase; CB1, cannabinoid receptor type 1; AT1, angiotensin receptor type 1; ACE, angiotensin converting enzyme.

There is no doubt that endocrine roles of adipose tissues, through adipokines, hormones, and other factors, significantly contribute to many aspects of the vascular system. Adipose tissue dysfunction is one of the major risk factors for cardiovascular diseases. In this regard, PVAT has a unique role due to its proximity to the blood vessel wall; thus, the significance of PVAT in modulating cardiovascular complications should not be ignored. During obesity and aging, dysfunctional PVAT promotes cardiovascular diseases via crosstalk with the underlying vascular cells, including the endothelium and VSMC under physiological and pathological conditions. Vascular oxidative stress is an important pathophysiological event in cardiometabolic complications of obesity, type 2 diabetes, and hypertension. Indeed, recent studies have suggested the potential use of antioxidants or targeting oxidative stress as novel therapeutic targets for PVAT-related cardiovascular complications (Figure 4).

Although the detailed mechanisms and targets are not completely known for antioxidant treatment in PVAT, there are two faces of ROS in adipose function and dysfunction. While ROS is needed as a secondary messenger of insulin signaling and vascular contractility, obesity-related oxidative stress can trigger the pathophysiology of the related cardiovascular diseases. It is important to maintain a physiological ROS level for adipogenic differentiation [171]. For example, a recent study has shown that antioxidant treatments with *N*-acetylcysteine, vitamin E, or glutathione ethyl ester may have a negative impact on oxygen consumption and cannot prevent ROS increase in adipose tissues [172]. Therefore, further research on potential antioxidant treatment targeting PVAT is needed. Reversing the shift of PVAT towards WAT-like during the development of obesity and aging might be a crucial strategy to maintaining a healthy vasculature. Even though this review focuses on the role of oxidative stress in vascular remodeling and endothelial function, the as-of-yet poorly understood secretome of PVAT (e.g., PVCF and PVRF) and their roles in the development of CVD should also be studied extensively. Future directions may fall into areas focusing on antioxidant treatments to promote PVAT function and restore browning. Detailed research is needed to dissect the molecular mechanisms of how oxidative stress modulates mitochondrial biogenesis in PVAT. SIRT1 and eNOS in PVAT are two important targets for enhancing PVAT function. Currently, there is still a lack of evidence dissecting the function of adipose SIRT1 and eNOS in mediating the browning processes of PVAT. Therefore, investigating the critical role of the interplay between PVAT SIRT1 and eNOS in controlling the browning and oxidative state of PVAT is of interest. Treatment with an antioxidant compound may be promising for restoring PVAT function during metabolic and cardiovascular diseases.
